# Medical Malpractice Stress Syndrome, Malpractice-Related Anxiety, and Defensive Medicine Practices Among Emergency Medicine Physicians in Türkiye: A National Cross-Sectional Survey

**DOI:** 10.3390/jcm15135098

**Published:** 2026-06-30

**Authors:** Hülya Yılmaz Başer, Aykut Başer, Sema Ayten, Melih Yucel Sanlier

**Affiliations:** 1 Department of Emergency Medicine, Faculty of Medicine, Bandırma Onyedi Eylül University, 10250 Bandırma, Turkey; hbaser@bandirma.edu.tr; 2 Department of Urology, Faculty of Medicine, Bandırma Onyedi Eylül University, 10250 Bandırma, Turkey; 3 Göztepe Education and Research Hospital, İstanbul Medeniyet University, 34722 Istanbul, Turkey; semayten@hotmail.com (S.A.); melihsanlier@hotmail.com (M.Y.S.)

**Keywords:** medical malpractice, defensive medicine, emergency medicine, anxiety, physician behavior, surveys and questionnaires, medical malpractice stress syndrome

## Abstract

**Background/Objectives**: Medical Malpractice Stress Syndrome (MMSS) describes the psychological and behavioral consequences of malpractice-related concerns and may contribute to defensive medicine practices. Emergency medicine physicians are particularly vulnerable to medicolegal risk because of the high-acuity and time-sensitive nature of their work. This study aimed to evaluate MMSS awareness, malpractice-related stress, and defensive medicine practices among emergency medicine specialists in Türkiye. **Methods**: A national cross-sectional web-based survey was conducted among emergency medicine specialists practicing in Türkiye between 1 April and 1 June 2026 and was distributed through professional emergency medicine networks and congress-related channels; because the survey was disseminated through multiple open professional platforms, an exact response rate could not be determined. The questionnaire assessed demographic characteristics, MMSS awareness, malpractice-related experiences and anxiety, and defensive medicine behaviors. Defensive medicine practices were evaluated using the validated Turkish version of the Defensive Medicine Attitude Scale. Associations between malpractice-related anxiety and defensive medicine behaviors were analyzed using Spearman correlation analysis. **Results**: A total of 128 emergency medicine specialists completed the survey. Malpractice-related anxiety was highly prevalent and frequently influenced professional attitudes and clinical decision-making. Defensive medicine practices were common, with assurance-type behaviors being substantially more frequent than avoidance-type behaviors. Malpractice-related anxiety demonstrated a significant positive correlation with assurance defensive medicine behaviors (r = 0.383, *p* < 0.001) but not with avoidance behaviors (r = 0.139, *p* = 0.118). Assurance and avoidance defensive medicine scores were positively correlated (r = 0.575, *p* < 0.001). **Conclusions**: MMSS and malpractice-related anxiety are highly prevalent among emergency medicine physicians in Türkiye and are associated with widespread defensive medicine practices. Malpractice-related anxiety appears to primarily promote assurance-type defensive medicine behaviors rather than avoidance behaviors. These findings suggest that medicolegal stress may influence clinical decision-making and contribute to increased healthcare utilization in emergency medicine.

## 1. Introduction

Medical malpractice remains a major concern for physicians worldwide and may influence both professional well-being and clinical decision-making. Beyond its legal and financial consequences, malpractice-related concerns can generate substantial psychological stress, potentially affecting physicians’ confidence, job satisfaction, and daily medical practice [1,2,3].

Emergency medicine is particularly vulnerable to medicolegal risk because physicians frequently make time-critical decisions under conditions of diagnostic uncertainty, high patient volume, limited clinical information, and overcrowding. Missed diagnoses, delayed treatment, communication failures, and systems-related factors are among the most common causes of malpractice claims in emergency care [4,5,6,7]. Consequently, emergency physicians are exposed not only to an elevated risk of litigation but also to considerable psychological pressure associated with potential adverse outcomes.

The psychological effects of malpractice concerns have been described as Medical Malpractice Stress Syndrome (MMSS), a condition characterized by anxiety, fear of litigation, reduced professional confidence, emotional distress, and behavioral changes in clinical practice [3,7,8]. Even in the absence of a formal lawsuit, the perception of medicolegal vulnerability may influence physicians’ attitudes and clinical decision-making. Previous studies have demonstrated that malpractice-related stress may affect physicians regardless of whether they have personally experienced litigation, suggesting that medicolegal concerns can influence the broader professional culture of healthcare practice [3,9].

One of the most important consequences of malpractice-related stress is defensive medicine. Defensive medicine refers to medical actions performed primarily to reduce perceived legal risk rather than to improve patient outcomes [10]. These behaviors may include ordering additional diagnostic tests, requesting consultations, increasing documentation, avoiding high-risk patients, or preferring less invasive management strategies [10]. A recent systematic review and meta-analysis by Zheng et al. reported a pooled prevalence of defensive medicine of 75.8% (95% CI: 72.3–79.3) among physicians worldwide, highlighting its global relevance and potential impact on healthcare systems [11].

In recent years, medicolegal issues have become increasingly visible within the Turkish healthcare system. Growing public awareness of patient rights, expanding complaint and reporting mechanisms, and increased media attention to malpractice-related cases have contributed to heightened medicolegal sensitivity among healthcare professionals. Recent evidence from Türkiye has also highlighted the continuing burden of malpractice claims and diagnostic errors in emergency departments, emphasizing the vulnerability of emergency physicians to medicolegal challenges [12]. Emergency medicine physicians may be particularly affected by these developments because they routinely manage undifferentiated patients under conditions of diagnostic uncertainty, time pressure, and overcrowding. Consequently, understanding the psychological and behavioral consequences of malpractice-related concerns has become increasingly relevant in contemporary emergency medicine practice.

Evidence from Türkiye suggests that medicolegal concerns are common among physicians working in high-risk specialties. Previous studies among emergency physicians, urologists, and obstetrician–gynecologists have demonstrated considerable levels of malpractice-related anxiety and defensive medical practices [3,13,14]. However, despite the inherently high-risk nature of emergency medicine, data regarding awareness of MMSS, malpractice-related stress, and defensive medicine practices among emergency medicine specialists in Türkiye remain limited.

To the best of our knowledge, no national study has comprehensively evaluated MMSS awareness, malpractice-related stress, and defensive medicine practices simultaneously among emergency medicine specialists in Türkiye. Therefore, the present study aimed to evaluate awareness of Medical Malpractice Stress Syndrome, assess the prevalence and characteristics of malpractice-related stress, and investigate defensive medicine practices among emergency medicine specialists in Türkiye. Additionally, we sought to explore the relationship between previous malpractice experiences, perceived medicolegal risk, and defensive clinical behaviors. We hypothesized that higher levels of malpractice-related anxiety would be associated with increased defensive medicine practices, particularly assurance-type defensive behaviors.

## 2. Materials and Methods

### 2.1. Study Design and Ethical Approval

This national cross-sectional survey was conducted among emergency medicine physicians actively practicing in Türkiye between 1 April 2026 and 1 June 2026. The study aimed to evaluate awareness of Medical Malpractice Stress Syndrome (MMSS), malpractice-related stress, and defensive medicine practices among emergency medicine physicians.

The study protocol was approved by the Non-Interventional Clinical Research Ethics Committee of Bandırma Onyedi Eylül University Faculty of Medicine (decision no. 2026-03-28, approved at meeting no. 2026-04, dated 25 March 2026). The study was conducted in accordance with the ethical principles of the Declaration of Helsinki. Electronic informed consent was obtained from all participants prior to survey access.

### 2.2. Participants and Survey Distribution

The target population consisted of emergency medicine specialists actively working in public hospitals, training and research hospitals, university hospitals, and private healthcare institutions throughout Türkiye.

The survey was distributed electronically through professional emergency medicine networks, social media platforms, e-mail groups, and physician messaging applications. The survey link was also disseminated through communication channels associated with the 22nd National Emergency Medicine Congress, which included approximately 1300 participants. Because the survey was distributed through multiple open professional networks and communication platforms, the exact number of eligible physicians who received or viewed the survey invitation could not be determined; therefore, an exact response rate could not be calculated. Participation was voluntary and anonymous, and no financial incentives were provided.

A total of 128 fully completed questionnaires were included in the final analysis. Given the voluntary and web-based nature of the survey, the achieved sample size (*n* = 128) was comparable to those reported in previous national surveys evaluating malpractice-related stress and defensive medicine among Turkish physicians, including studies conducted among urologists (*n* = 152) and obstetrician–gynecologists (*n* = 127) [3,14].

To improve the quality and reporting of the web-based survey, the Checklist for Reporting Results of Internet E-Surveys (CHERRIES) guidelines were followed throughout the study design, implementation, and reporting processes [15].

### 2.3. Questionnaire Development

The questionnaire was developed following a comprehensive review of the literature regarding medical malpractice, Medical Malpractice Stress Syndrome (MMSS), and defensive medicine practices [3,10,11,14]. The survey structure was adapted from previously published national studies evaluating MMSS and defensive medicine behaviors among urologists and obstetrician–gynecologists in Türkiye [3,14].

The final questionnaire consisted of 41 items organized into three sections.

The first section collected demographic and professional characteristics, including age, institution type, academic title, years of professional experience, annual patient volume, and previous exposure to malpractice investigations or litigation.

The second section evaluated participants’ awareness of MMSS, previous malpractice experiences, malpractice-related anxiety, perceived medicolegal vulnerability, professional consequences of malpractice concerns, and potential sources of support. Several items in this section were adapted from the previously published MMSS questionnaire developed for Turkish urologists [3].

The third section evaluated defensive medicine practices using the Turkish version of the Defensive Medicine Attitude Scale developed and validated by Kolcu et al. [16]. The defensive medicine items derived from this validated instrument were applied without modification. Additional questionnaire sections evaluating MMSS awareness, malpractice-related experiences, and demographic characteristics were developed separately based on previously published national studies and the relevant literature [3,14]. The scale includes items evaluating both assurance behaviors (e.g., additional testing, imaging, consultations, documentation, and informed consent practices) and avoidance behaviors (e.g., avoidance of high-risk patients, complex cases, and invasive procedures) [16].

In addition to individual item analyses, composite assurance and avoidance defensive medicine scores were derived to facilitate quantitative assessment of defensive medicine tendencies.

Survey items were presented using multiple-choice and five-point Likert-type response formats, where appropriate. The estimated time required to complete the questionnaire was approximately 10–15 min.

### 2.4. Data Quality Control

The survey was created using Google Forms^®^ (Google LLC, Mountain View, CA, USA). All mandatory questions required completion before progression to subsequent sections, thereby minimizing missing data. Only fully completed questionnaires were included in the final analysis. Duplicate responses were minimized by restricting participation to a single submission per respondent. No identifying personal information was collected, ensuring participant anonymity and confidentiality. Because all survey items were configured as mandatory fields within the Google Forms platform, partial item non-response was not possible. Only fully completed questionnaires could be submitted and included in the final analysis.

### 2.5. Defensive Medicine Composite Scores

To facilitate quantitative analysis of defensive medicine practices, questionnaire items were grouped into two conceptual domains: assurance behaviors and avoidance behaviors.

Assurance behaviors included increased use of diagnostic tests and imaging studies, more frequent consultation requests, enhanced documentation practices, greater emphasis on informed consent and patient information, spending more time with patients, recommending hospital admission beyond strict clinical indications, and the influence of malpractice-related media coverage on clinical practice.

Avoidance behaviors included avoiding patients perceived to have a high likelihood of litigation, avoiding patients with complex medical conditions, avoiding treatments associated with a high risk of complications, and preferring non-invasive approaches over invasive alternatives.

Responses were originally recorded on a five-point Likert scale ranging from 1 (“strongly agree”) to 5 (“strongly disagree”). Prior to score calculation, all items were reverse-coded so that higher scores reflected a greater tendency toward defensive medicine practices.

The Assurance Defensive Medicine Score was calculated by summing responses to nine assurance-related items (possible range: 9–45), whereas the Avoidance Defensive Medicine Score was calculated by summing responses to four avoidance-related items (possible range: 4–20).

One questionnaire item assessing whether participants prescribed medications strictly within clinical indications was analyzed separately and was not incorporated into either composite score because it did not conceptually represent either assurance-type or avoidance-type defensive medicine behavior.

### 2.6. Statistical Analysis

Statistical analyses were performed using IBM SPSS Statistics for Windows, Version 22.0 (IBM Corp., Armonk, NY, USA). Categorical variables were presented as frequencies and percentages, whereas continuous variables were expressed as mean ± standard deviation (SD). Associations between categorical variables were evaluated using Pearson’s chi-square test. Fisher’s exact test was applied when expected cell counts were insufficient for chi-square analysis.

Correlations between malpractice-related anxiety and defensive medicine composite scores were assessed using Spearman’s rank correlation coefficient. The internal consistency of the Assurance Defensive Medicine Score and Avoidance Defensive Medicine Score was evaluated using Cronbach’s alpha coefficient. Cronbach’s alpha coefficients ≥0.70 were considered indicative of acceptable internal consistency. All statistical tests were two-tailed, and a *p* value < 0.05 was considered statistically significant.

## 3. Results

### 3.1. Participant Characteristics

A total of 128 emergency medicine physicians completed the survey and were included in the final analysis. The demographic and professional characteristics of the participants are presented in Table 1. More than half of the respondents were aged 25–39 years (54.7%, *n* = 70), while 32.0% (*n* = 41) were aged 40–49 years and 13.3% (*n* = 17) were aged 50–59 years. The most common professional experience category was 11–15 years (34.4%, *n* = 44), followed by 16–20 years (26.6%, *n* = 34) and 0–5 years (23.4%, *n* = 30).

Most participants were specialist physicians (74.2%, *n* = 95), whereas 14.8% (*n* = 19) were associate professors and 10.9% (*n* = 14) were assistant professors. Regarding workplace characteristics, the majority were employed in training and research hospitals (57.8%, *n* = 74), followed by university hospitals (28.9%, *n* = 37) and state hospitals (13.3%, *n* = 17). Nearly one-third of respondents reported managing 1001–5000 patients annually (28.9%, *n* = 37), whereas 35.9% reported caring for more than 10,000 patients per year.

### 3.2. Awareness of MMSS, Malpractice Exposure, and Defensive Medicine

Participants demonstrated substantial exposure to malpractice-related events and concerns (Table 2). Although only 16.4% (*n* = 21) reported having personally been involved in a malpractice lawsuit, 78.1% (*n* = 100) indicated that a colleague had previously faced malpractice litigation. Nearly all respondents (98.4%, *n* = 126) believed that malpractice lawsuits negatively affect physician performance.

Awareness of defensive medicine was high among participants, with 96.1% (*n* = 123) reporting familiarity with the concept. However, only 57.0% (*n* = 73) considered themselves sufficiently knowledgeable about defensive medicine practices. In contrast, awareness of Medical Malpractice Stress Syndrome (MMSS) was considerably lower, with only 29.7% (*n* = 38) reporting prior knowledge of the concept, whereas 70.3% (*n* = 90) had never heard of MMSS.

### 3.3. Malpractice-Related Anxiety and Its Professional Consequences

Malpractice-related anxiety was common among participants (Table 3). Overall, 84.4% of respondents reported at least some degree of fear or anxiety about being involved in malpractice litigation. Anxiety was reported as frequent by 39.1% (*n* = 50) and constant by 26.6% (*n* = 34), whereas 15.6% (*n* = 20) reported no malpractice-related fear or anxiety.

When participants were asked how often the possibility of malpractice litigation came to mind during the previous month, 50.1% (*n* = 64) reported thinking about it 1–3 days per month, 13.3% (*n* = 17) reported 1–2 days per week, 14.1% (*n* = 18) reported 3–5 days per week, and 7.0% (*n* = 9) reported thinking about it almost every day. Among participants who reported malpractice-related anxiety (*n* = 128), these thoughts most commonly occurred while caring for yellow-zone patients (36%, *n* = 73), followed by red-zone patients (32%, *n* = 65), green-zone patients (20.2%, *n* = 41), and during non-working hours or private life (11.8%, *n* = 24).

Malpractice-related concerns also appeared to influence professional attitudes and clinical decision-making. Overall, 20.3% of participants (*n* = 26) reported having considered leaving the profession because of these concerns, while 76.6% (*n* = 98) stated that they had reconsidered at least one medical decision due to the possibility of malpractice litigation.

### 3.4. Perceived Sources of Medicolegal Risk and Support Systems

**Figure 1:**
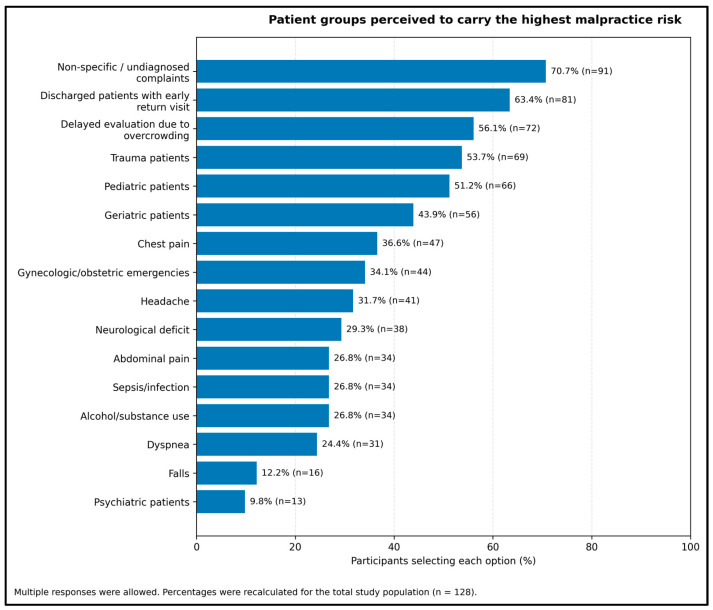
Patient groups perceived to carry the highest risk of malpractice litigation among emergency medicine physicians in Türkiye.

The patient groups perceived to be at the highest risk for malpractice litigation are presented in Figure 1. The most frequently identified group was patients presenting with non-specific or undiagnosed complaints (70.7%, *n* = 91), followed by patients who re-presented shortly after discharge (63.4%, *n* = 81) and patients whose evaluation was delayed because of emergency department overcrowding (56.1%, *n* = 72). Trauma patients (53.7%, *n* = 69) and pediatric patients (51.2%, *n* = 66) were also commonly perceived as carrying an elevated medicolegal risk. In contrast, psychiatric patients (9.8%, *n* = 13) and patients presenting after falls (12.2%, *n* = 16) were less frequently identified as high-risk groups (Figure 1).

**Figure 2:**
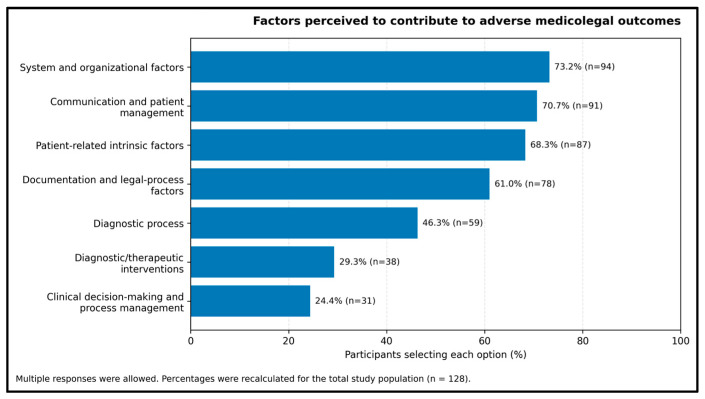
Factors perceived to contribute to adverse medicolegal outcomes in high-risk patient groups.

Participants were also asked to identify factors contributing to adverse medicolegal outcomes in these patient populations (Figure 2). System and organizational factors were the most frequently reported contributors (73.2%, *n* = 94), followed by communication and patient-management issues (70.7%, *n* = 91), patient-related intrinsic factors (68.3%, *n* = 87), and documentation and legal-process-related factors (61.0%, *n* = 78). Diagnostic processes were selected by 46.3% (*n* = 59) of respondents, whereas diagnostic and therapeutic interventions (29.3%, *n* = 38) and clinical decision-making and process management (24.4%, *n* = 31) were less frequently reported.

**Figure 3:**
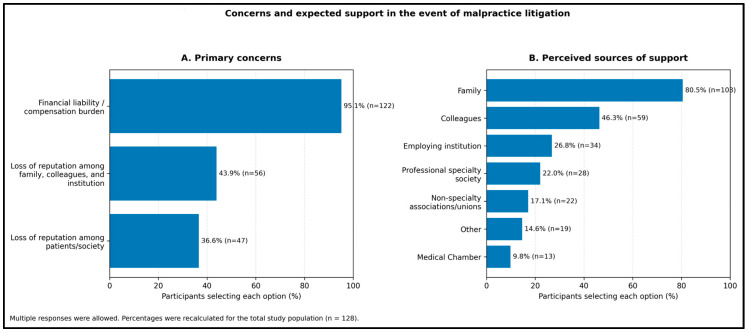
Concerns and perceived support systems in the event of malpractice litigation. (**A**) Primary concerns associated with potential malpractice litigation. Financial liability and compensation burden were the most frequently reported concerns, followed by concerns regarding professional and social reputation. (**B**) Perceived sources of support following malpractice litigation. Family and colleagues were the most commonly identified sources of support, whereas institutional and professional organizations were less frequently perceived as supportive resources. Bars represent the proportion of participants selecting each response option. Multiple responses were allowed.

The principal concerns associated with potential malpractice litigation are presented in Figure 3A. Financial liability and compensation burden emerged as the dominant concern, reported by 95.1% of participants (*n* = 122). Concerns regarding loss of reputation among family members, colleagues, and within the workplace were reported by 43.9% (*n* = 56), while 36.6% (*n* = 47) expressed concern about loss of reputation in the eyes of patients and society.

Perceived sources of support following malpractice litigation are summarized in Figure 3B. Family was identified as the most important source of support (80.5%, *n* = 103), followed by colleagues (46.3%, *n* = 59). In contrast, institutional and professional support systems were reported less frequently. Only 26.8% (*n* = 34) of respondents expected support from their employing institution, 22.0% (*n* = 28) from their specialty society, and 9.8% (*n* = 13) from the Medical Chamber.

### 3.5. Defensive Medicine Practices

**Figure 4:**
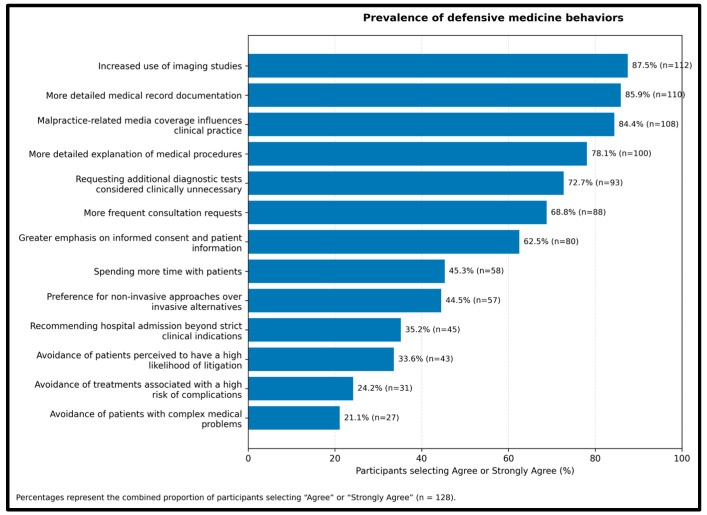
Prevalence of defensive medicine practices among emergency medicine physicians in Türkiye. Bars represent the proportion of participants reporting agreement or strong agreement with each defensive medicine behavior.

The prevalence of defensive medicine practices is presented in Table 4 and Figure 4. Overall, assurance-type defensive behaviors were substantially more common than avoidance-type behaviors.

Among assurance behaviors, participants most frequently reported increased utilization of diagnostic resources and documentation practices. More than four-fifths of respondents indicated that malpractice-related concerns influenced their use of imaging studies and medical record documentation. Similarly, a considerable proportion reported requesting additional diagnostic tests, increasing consultation requests, and providing more detailed explanations regarding medical procedures and potential complications. More than half of the participants also reported placing greater emphasis on informed consent and patient information processes.

In contrast, avoidance-type behaviors were less frequently reported. Approximately one-third of respondents indicated that they avoided patients perceived to have a high likelihood of litigation, whereas avoidance of patients with complex medical conditions and avoidance of treatments associated with a high risk of complications were considerably less common. Likewise, fewer than half of the participants reported preferring non-invasive management strategies over invasive alternatives.

Notably, malpractice-related concerns appeared to influence not only diagnostic and therapeutic decision-making but also broader professional attitudes. A substantial majority of respondents reported that malpractice-related news and media coverage affected their clinical practice. In addition, more than one-third acknowledged recommending hospital admission for reasons extending beyond strict clinical indications.

### 3.6. Factors Associated with Reconsidering Medical Decisions Due to Malpractice Concerns

Factors associated with reconsidering medical decisions due to malpractice concerns are summarized in Table 5. Overall, 76.6% of participants (*n* = 98) reported that they had reconsidered at least one medical decision because of the possibility of malpractice litigation.

A history of malpractice litigation involving a colleague was significantly associated with reconsidering medical decisions. Participants who reported that a colleague had faced malpractice litigation were more likely to reconsider their clinical decisions than those without such exposure (83.0% vs. 53.6%, *p* = 0.002). Malpractice-related fear/anxiety was also significantly associated with reconsidering clinical decisions (*p* = 0.001), with the highest proportion observed among participants who reported constant malpractice-related anxiety.

Academic title and annual patient volume were also significantly associated with reconsidering medical decisions. Specialist physicians reported reconsidering medical decisions more frequently than assistant professors and associate professors (84.2%, 35.7%, and 68.4%, respectively; *p* < 0.001). Similarly, the frequency of reconsidering medical decisions differed significantly across annual patient volume categories (*p* < 0.001).

In contrast, age group, institution type, professional experience, awareness of MMSS, and self-reported knowledge of defensive medicine were not significantly associated with reconsidering medical decisions due to malpractice concerns. Personal malpractice lawsuit experience showed a statistically significant association; however, participants with personal litigation experience reported reconsidering decisions less frequently than those without such experience (57.1% vs. 80.4%, *p* = 0.045), and this finding should be interpreted cautiously because of the small number of participants with personal lawsuit experience.

### 3.7. Defensive Medicine Composite Scores and Their Association with Malpractice-Related Anxiety

To facilitate quantitative analysis of defensive medicine practices, two composite scores were generated. The Assurance Defensive Medicine Score consisted of nine assurance-related defensive medicine items, whereas the Avoidance Defensive Medicine Score consisted of four avoidance-related items. Both scores demonstrated good internal consistency, with Cronbach’s alpha coefficients of 0.776 and 0.775, respectively.

The descriptive characteristics of the composite scores are presented in Table 6. The mean Assurance Defensive Medicine Score was 33.83 ± 5.21 (range: 21–43), indicating a relatively high prevalence of assurance-type defensive medicine behaviors among participants. The mean Avoidance Defensive Medicine Score was 11.00 ± 3.60 (range: 4–19), suggesting a comparatively lower but still substantial tendency toward avoidance-type defensive medicine practices.

The association between malpractice-related fear/anxiety and defensive medicine composite scores was evaluated using Spearman correlation analysis (Table 7). A significant positive correlation was observed between malpractice-related fear/anxiety and the Assurance Defensive Medicine Score (r = 0.383, *p* < 0.001). Physicians reporting higher levels of malpractice-related anxiety were more likely to engage in assurance-type defensive medicine behaviors, including increased use of diagnostic tests and imaging studies, more frequent consultation requests, enhanced documentation practices, and greater emphasis on informed consent procedures.

In contrast, no significant correlation was observed between malpractice-related fear/anxiety and the Avoidance Defensive Medicine Score (r = 0.139, *p* = 0.118). This finding suggests that malpractice-related anxiety primarily influences behaviors aimed at increasing diagnostic certainty and medicolegal protection rather than behaviors involving avoidance of patients, procedures, or treatments.

A significant positive correlation was also observed between the Assurance and Avoidance Defensive Medicine Scores (r = 0.575, *p* < 0.001), indicating that physicians who reported greater engagement in one type of defensive medicine behavior also tended to report greater engagement in the other.

## 4. Discussion

### 4.1. Principal Findings

This study is, to our knowledge, the first national survey to evaluate Medical Malpractice Stress Syndrome (MMSS), malpractice-related anxiety, and defensive medicine practices simultaneously among emergency medicine physicians in Türkiye. The main findings were that malpractice-related anxiety was common, awareness of MMSS was relatively low, defensive medicine practices were widespread, and assurance-type behaviors were more prominent than avoidance-type behaviors. Most importantly, malpractice-related anxiety was significantly associated with assurance defensive medicine behaviors, but not with avoidance behaviors.

These findings are consistent with previous evidence indicating that emergency medicine is a specialty with substantial medicolegal vulnerability. Emergency physicians routinely care for undifferentiated patients under conditions of diagnostic uncertainty, time pressure, overcrowding, and limited clinical information, all of which may increase perceived litigation risk and influence clinical decision-making [4,5,6,7,9].

Our findings also align with previous studies from Türkiye showing that malpractice-related stress and defensive medicine are important concerns in high-risk specialties, including urology and obstetrics and gynecology [3,14]. However, emergency medicine differs from many procedural specialties because physicians usually cannot select patients, defer initial evaluation, or avoid high-risk encounters. This feature may help explain why malpractice-related anxiety in our cohort was expressed mainly through assurance-type defensive behaviors rather than avoidance behaviors.

Overall, the present findings suggest that malpractice-related stress is not only a legal concern but also an occupational and behavioral issue that may shape clinical practice in emergency departments. Although direct comparisons should be interpreted cautiously because of differences in study populations and survey methodologies, the high prevalence of malpractice-related anxiety observed in the present study appears broadly consistent with previous Turkish studies conducted among urologists and obstetrician–gynecologists, suggesting that MMSS-related concerns are common across multiple high-risk medical specialties in Türkiye [3,14].

### 4.2. Malpractice-Related Anxiety and Professional Consequences

One of the most important findings of the present study was the high prevalence of malpractice-related fear and anxiety among emergency medicine physicians. Although awareness of Medical Malpractice Stress Syndrome (MMSS) was relatively low, malpractice-related concerns were widespread and appeared to influence professional attitudes and clinical decision-making. This finding supports the view that malpractice-related stress represents an important occupational burden in emergency medicine, extending beyond the direct legal consequences of litigation [4,5,6,7].

Emergency medicine is particularly vulnerable to malpractice-related anxiety because physicians are required to make rapid diagnostic and therapeutic decisions under conditions of uncertainty, incomplete information, time pressure, overcrowding, and high patient volume. Previous studies have shown that emergency physicians are frequently exposed to malpractice claims and medicolegal scrutiny [4,5,6,7,9]. In Türkiye, Başer and Akın reported that emergency departments represent a high-risk environment for malpractice claims involving negligent homicide, with failure to conduct adequate medical evaluation being the most common reason for malpractice allegations [12]. These findings are consistent with our results, in which participants identified non-specific or undiagnosed complaints, re-presentations shortly after discharge, and overcrowding-related delays as major sources of medicolegal risk.

An important observation in our study was that malpractice-related concerns were not limited to physicians with personal litigation experience. Exposure to malpractice litigation involving colleagues was significantly associated with reconsideration of medical decisions. Similar findings were reported among Turkish urologists, suggesting that indirect exposure to medicolegal events may influence physicians’ perceptions and behaviors even in the absence of personal litigation [3]. This may reflect a broader professional climate in which adverse outcomes, complaints, and lawsuits are shared within clinical networks and contribute to collective medicolegal anxiety. The consistency of these findings across different specialties may indicate that malpractice-related stress is influenced not only by specialty-specific clinical risks but also by broader medicolegal and organizational factors within the healthcare system.

Our findings are also supported by earlier emergency medicine literature. Rodriguez et al. demonstrated that malpractice concern was already present among emergency medicine residents at the beginning of training and did not significantly disappear by graduation, suggesting that fear of litigation may become embedded early in emergency medicine professional development [17]. More recently, Şahin et al. reported that fear of malpractice among emergency medicine residents in Türkiye was related to procedural anxiety and perceived clinical competence, emphasizing the interaction between medicolegal concerns, clinical confidence, and performance in high-risk emergency procedures [18].

The professional consequences of malpractice-related stress are clinically important. In the present study, more than three-quarters of participants reported reconsidering at least one medical decision because of malpractice concerns, and one-fifth had considered leaving the profession. Although caution may contribute to safer decision-making in some contexts, excessive medicolegal concern may increase uncertainty, reinforce defensive behaviors, and shift decision-making from patient-centered reasoning toward self-protective practice.

Taken together, these findings suggest that malpractice-related anxiety among emergency medicine physicians should be considered not only a legal issue but also an occupational health and workforce sustainability concern. Educational programs, institutional support systems, peer-support mechanisms, and medicolegal risk-management training may help reduce the professional burden associated with malpractice-related stress.

### 4.3. Institutional and Psychosocial Dimensions of Malpractice Stress

An important contribution of the present study is the demonstration that malpractice-related stress extends beyond concerns about legal liability and encompasses broader psychosocial and institutional dimensions. Participants reported substantial concern regarding financial consequences, reputational damage, media exposure, and professional uncertainty. These findings suggest that the burden of malpractice-related stress cannot be fully explained by the risk of litigation alone but should instead be viewed as a multidimensional occupational stressor.

The financial consequences of malpractice allegations represented the most frequently reported concern in our cohort. This observation is consistent with previous studies conducted among Turkish physicians, which have demonstrated that the perceived economic burden of litigation is often accompanied by concerns regarding professional reputation, career progression, and public perception [3,14]. Similar concerns have also been reported internationally, where physicians frequently identify reputational harm and public scrutiny as consequences that may be as distressing as the legal process itself [19].

Another notable finding was the prominent role of informal support systems. Most participants reported that they would primarily seek support from family members, friends, or colleagues rather than from institutional structures. In contrast, relatively few participants identified hospitals, professional organizations, or legal advisory services as potential sources of support. This finding may reflect limited confidence in existing institutional support mechanisms following adverse events or medicolegal investigations.

Recent evidence from emergency medicine supports this interpretation. Şahin et al. reported that emergency medicine residents frequently rely on supervision and informal peer support when managing malpractice-related concerns and procedural anxiety, highlighting the importance of professional mentorship and collegial support in high-risk clinical environments [18]. Together with our findings, these results suggest that interpersonal support networks may currently play a greater role than formal institutional mechanisms in mitigating malpractice-related stress.

The influence of media coverage on clinical behavior observed in our study also deserves attention. Contemporary literature suggests that physicians increasingly perceive malpractice not only as a legal risk but also as a reputational and social risk. In a systematic review of European literature, Baungaard et al. demonstrated that modern definitions of defensive medicine extend beyond fear of litigation and include concerns regarding criticism, complaints, reputational damage, and professional scrutiny [19]. This broader conceptualization may help explain why media-related malpractice coverage was reported to influence clinical decision-making among a substantial proportion of participants in our study.

Taken together, these findings indicate that malpractice-related stress should be understood as a complex phenomenon involving legal, psychological, social, and institutional components. Efforts aimed at reducing malpractice-related stress may therefore require not only medicolegal reforms but also stronger organizational support systems, peer-support programs, mentoring structures, and physician wellness initiatives.

### 4.4. Defensive Medicine Practices Among Emergency Physicians

Defensive medicine behaviors were highly prevalent among emergency medicine physicians in the present study. The most frequently reported practices included increased use of diagnostic imaging, more frequent consultation requests, enhanced documentation, greater emphasis on informed consent, and additional diagnostic testing. These findings suggest that defensive medicine remains a common component of everyday clinical decision-making in emergency departments.

Our results are consistent with previous national and international studies demonstrating widespread defensive medicine practices among physicians working in high-risk specialties. In a national survey of Turkish urologists, malpractice-related concerns were associated with increased use of diagnostic testing, consultation requests, and documentation practices [3]. Similarly, Gunenc et al. reported substantial levels of defensive medicine behaviors among obstetricians and gynecologists in Türkiye [14]. Internationally, defensive medicine has been reported across a broad range of specialties and healthcare systems, suggesting that medicolegal concerns represent a global driver of physician behavior [1,10,11].

A recent systematic review and meta-analysis by Zheng et al. estimated that approximately three-quarters of physicians worldwide engage in some form of defensive medicine practice [11]. Although direct comparisons between studies should be interpreted cautiously because of differences in measurement tools and healthcare systems, our findings are consistent with the broader literature indicating that defensive medicine has become a common response to perceived medicolegal risk.

An important observation in our study was the predominance of assurance-type defensive medicine behaviors. Physicians were considerably more likely to report ordering additional investigations, requesting consultations, increasing documentation, or strengthening informed consent procedures than avoiding patients or declining high-risk clinical situations. This pattern is particularly relevant in emergency medicine, where opportunities to avoid patient encounters are inherently limited. Emergency physicians are generally required to evaluate all presenting patients regardless of clinical complexity, perceived litigation risk, or diagnostic uncertainty.

This interpretation is supported by previous malpractice research in emergency medicine. Diagnostic uncertainty and concerns regarding missed diagnoses remain among the most important drivers of malpractice claims in emergency departments [5,12]. Consequently, physicians may respond to medicolegal concerns by seeking greater diagnostic certainty through additional testing and consultation rather than by avoiding patient care. Such behaviors may be perceived as protective strategies aimed at reducing clinical uncertainty and legal vulnerability.

Contemporary literature also suggests that defensive medicine extends beyond purely legal concerns. Baungaard et al. demonstrated that modern conceptualizations of defensive medicine increasingly incorporate reputational concerns, complaints, criticism, and professional scrutiny in addition to traditional litigation fears [19]. This broader perspective may help explain why defensive medicine behaviors remain common even among physicians who have never personally experienced a malpractice lawsuit.

An additional noteworthy finding was the moderate-to-strong positive correlation between assurance and avoidance defensive medicine scores. Although assurance and avoidance behaviors are often conceptualized as distinct dimensions of defensive medicine, our findings suggest that they may coexist within the same physicians. In other words, physicians who frequently engage in assurance-type behaviors, such as increased testing, documentation, and consultation requests, may also be more likely to adopt avoidance-oriented behaviors in selected clinical situations. This observation supports the growing view that defensive medicine represents a broader medicolegal coping strategy rather than two entirely independent behavioral patterns. Similar observations have been reported in previous studies, indicating that physicians exposed to greater medicolegal stress often adopt multiple defensive strategies simultaneously rather than relying on a single behavioral response [10,11,19]. This finding may be particularly relevant in emergency medicine, where physicians face continuous exposure to diagnostic uncertainty, time pressure, and potential medicolegal scrutiny. Under such conditions, multiple defensive responses may emerge concurrently rather than as isolated behaviors.

Taken together, our findings suggest that defensive medicine among emergency physicians is driven by a combination of medicolegal, diagnostic, organizational, and reputational factors. While some defensive behaviors may contribute to perceived diagnostic safety, excessive reliance on unnecessary testing, consultation, and documentation may increase healthcare costs, contribute to resource utilization, and potentially affect the efficiency of emergency care delivery. Previous studies have suggested that defensive medicine may represent a substantial economic burden for healthcare systems, particularly in high-risk specialties where advanced imaging, specialist consultations, and precautionary admissions are frequently utilized [10,11]. Although the present study was not designed to quantify healthcare expenditures directly, the high prevalence of assurance-type defensive medicine behaviors observed among participants suggests that malpractice-related anxiety may influence not only physician decision-making but also healthcare resource allocation and system-level efficiency.

### 4.5. Relationship Between Malpractice-Related Anxiety and Defensive Medicine

One of the most important findings of this study was the differential relationship between malpractice-related anxiety and defensive medicine behaviors. While malpractice-related anxiety demonstrated a significant positive correlation with the Assurance Defensive Medicine Score, no significant association was observed with the Avoidance Defensive Medicine Score. These findings suggest that emergency physicians primarily respond to medicolegal concerns by increasing protective clinical behaviors rather than by avoiding high-risk patients or clinical situations.

This observation is consistent with the unique characteristics of emergency medicine practice. Unlike many elective or procedural specialties, emergency physicians generally cannot choose their patients, postpone evaluations, or avoid clinically complex presentations. Consequently, avoidance-type defensive medicine behaviors may be less feasible in emergency settings. Instead, physicians may attempt to reduce perceived medicolegal vulnerability by increasing diagnostic testing, consultation requests, documentation practices, and informed consent discussions.

Our findings are broadly consistent with the classical framework proposed by Studdert et al., who distinguished assurance and avoidance forms of defensive medicine and demonstrated that physicians frequently employ reassurance-oriented strategies in response to malpractice concerns [10]. Similarly, previous emergency medicine literature has suggested that fear of litigation often promotes increased diagnostic testing and consultation rather than complete avoidance of patient care [17]. The predominance of assurance behaviors observed in our study appears to support this interpretation.

An additional explanation may be provided by contemporary conceptualizations of defensive medicine. Baungaard et al. argued that defensive medical behavior should not be viewed solely as a response to litigation risk but also as a response to professional scrutiny, criticism, complaints, reputational concerns, and uncertainty [19]. Within this framework, assurance behaviors may serve not only to reduce legal exposure but also to provide physicians with a greater sense of professional security and diagnostic confidence. This may be particularly relevant in emergency medicine, where clinical decisions are frequently made under conditions of uncertainty and time pressure.

Interestingly, assurance and avoidance defensive medicine scores were moderately correlated with each other, suggesting that these behaviors may not represent entirely independent constructs. Physicians who exhibit greater assurance-type behaviors may also demonstrate some degree of avoidance tendency. However, only assurance behaviors showed a direct relationship with malpractice-related anxiety, indicating that reassurance-oriented strategies may represent the primary behavioral manifestation of malpractice stress among emergency physicians.

Taken together, these findings suggest that malpractice-related anxiety influences how emergency physicians practice rather than whether they engage with high-risk patients. The predominance of assurance-type defensive medicine may reflect an adaptive attempt to increase diagnostic certainty and reduce perceived medicolegal risk. Nevertheless, excessive reliance on such behaviors may contribute to unnecessary testing, increased healthcare expenditures, and inefficient resource utilization. Future prospective studies are needed to determine whether interventions aimed at reducing malpractice-related stress can also reduce defensive medicine behaviors without compromising patient safety.

### 4.6. Strengths and Limitations

This study has several limitations that should be considered when interpreting the findings. First, the cross-sectional design precludes causal inference regarding the relationship between malpractice-related anxiety and defensive medicine behaviors. Second, the study relied on self-reported responses, which may be influenced by recall bias and social desirability bias. Participants may have been less likely to acknowledge avoidance-type defensive medicine behaviors perceived as professionally undesirable, potentially resulting in an underestimation of avoidance practices relative to assurance-type behaviors. Third, participation was voluntary and conducted through a web-based survey, introducing the possibility of selection bias and limiting control over non-response characteristics. In addition, although the sample size was comparable to those reported in previous physician surveys investigating malpractice-related stress and defensive medicine in Türkiye and internationally [3,14], the study population represents only a proportion of emergency medicine physicians practicing nationally. Furthermore, although the present study identified widespread assurance-type defensive medicine behaviors, it was not designed to quantify their direct economic impact. Consequently, the potential effects of defensive medicine on healthcare expenditures, resource utilization, and system-level costs could not be evaluated. Therefore, caution is warranted when generalizing the findings to all emergency physicians in Türkiye.

Nevertheless, several factors strengthen the validity of the present study. To our knowledge, this is the first national study specifically evaluating Medical Malpractice Stress Syndrome, malpractice-related anxiety, and defensive medicine practices simultaneously among emergency medicine physicians in Türkiye. The study employed a structured questionnaire derived from previously published instruments, followed CHERRIES recommendations for web-based surveys [15], and incorporated both descriptive and quantitative analyses of defensive medicine behaviors. Furthermore, the inclusion of composite assurance and avoidance defensive medicine scores allowed a more detailed evaluation of the relationship between malpractice-related anxiety and different defensive medicine strategies. Taken together, these strengths provide a comprehensive overview of malpractice-related stress and defensive medicine behaviors in Turkish emergency medicine practice and may serve as a foundation for future multicenter and longitudinal investigations.

## 5. Conclusions

Medical Malpractice Stress Syndrome awareness remains limited among emergency medicine physicians in Türkiye, despite the high prevalence of malpractice-related anxiety and medicolegal concerns. Defensive medicine practices were common, with assurance-type behaviors such as increased diagnostic testing, consultation requests, documentation, and informed consent practices being substantially more prevalent than avoidance-type behaviors.

A key finding of this study is that malpractice-related anxiety was significantly associated with assurance defensive medicine behaviors but not with avoidance behaviors. This suggests that emergency physicians primarily respond to medicolegal concerns by seeking greater diagnostic certainty and professional protection rather than by avoiding high-risk patients or clinical situations. The findings further indicate that malpractice-related stress extends beyond legal liability and encompasses important psychological, professional, social, and institutional dimensions. Financial concerns, perceived medicolegal vulnerability, media influence, and limited reliance on formal institutional support mechanisms may all contribute to physicians’ experiences of malpractice-related stress.

Efforts to reduce malpractice-related stress should therefore extend beyond legal reforms and include physician education, organizational support systems, peer-support programs, and medicolegal risk-management training. Such interventions may improve physician well-being while promoting more efficient and sustainable emergency care delivery.

Future multicenter and prospective studies are warranted to further clarify the long-term impact of malpractice-related stress on physician behavior, healthcare utilization, and patient outcomes.

## Figures and Tables

**Table 1:** Demographic and professional characteristics of the study participants (*n* = 128).

Variable	Category	*n* (%)
Age (years)	25–39	70 (54.7)
	40–49	41 (32.0)
	50–59	17 (13.3)
Professional experience	0–5 years	30 (23.4)
	6–10 years	15 (11.7)
	11–15 years	44 (34.4)
	16–20 years	34 (26.6)
	>20 years	5 (3.9)
Academic title	Specialist Physician	95 (74.2)
	Assistant Professor	14 (10.9)
	Associate Professor	19 (14.8)
Institution type	State Hospital	17 (13.3)
	Training and Research Hospital	74 (57.8)
	University Hospital	37 (29.9)
Annual patient volume	0–1000	17 (13.3)
	1001–5000	37 (28.9)
	5001–10,000	28 (21.9)
	10,001–15,000	21 (16.4)
	>15,000	25 (19.5)

**Table 2:** Awareness of medical malpractice, MMSS, and defensive medicine among participants (*n* = 128).

Variable	Category	*n* (%)
Previous malpractice lawsuit against participant	No	107 (83.6)
	Yes	21 (16.4)
Malpractice lawsuit against a colleague	No	28 (21.9)
	Yes	100 (78.1)
Malpractice litigation affects physician performance	No	2 (1.6)
	Yes	126 (98.4)
Heard of the concept of defensive medicine	No	5 (3.9)
	Yes	123 (96.1)
Adequate knowledge regarding defensive medicine	No	29 (22.7)
	Undecided	26 (20.3)
	Yes	73 (57.0)
Heard of the concept of Medical Malpractice Stress Syndrome (MMSS)	No	90 (70.3)
	Yes	38 (29.7)

**Table 3:** Malpractice-related anxiety and its professional consequences among emergency medicine physicians (*n* = 128).

Variable	Category	*n* (%)
Malpractice-related fear/anxiety	No fear/anxiety (0%)	20 (15.6)
	Rarely (10–30%)	5 (3.9)
	Sometimes (40–60%)	19 (14.8)
	Frequently (70–90%)	50 (39.1)
	Always (100%)	34 (26.6)
Frequency of thinking about malpractice litigation during the previous month	Never (0 days)	20 (15.6)
	1–3 days/month	64 (50.0)
	1–2 days/week	17 (13.3)
	3–5 days/week	18 (14.1)
	Almost every day	9 (7.0)
Situations in which malpractice-related thoughts occur *	Yellow-zone patient care	73 (36.0)
	Red-zone patient care	65 (32.0)
	Green-zone patient care	41 (20.2)
	Outside work/private life	24 (11.8)
Considered leaving the profession because of malpractice concerns	No	102 (79.7)
	Yes	26 (20.3)
Reconsidered a medical decision because of malpractice concerns	No	30 (23.4)
	Yes	98 (76.6)

* Multiple responses were allowed. Percentages were calculated among participants reporting malpractice-related fear or anxiety (*n* = 128).

**Table 4:** Prevalence of defensive medicine practices among emergency medicine physicians (*n* = 128).

Defensive Medicine Behavior	*n* (%)
Increased use of imaging studies	112 (87.5)
More detailed medical record documentation	110 (85.9)
Malpractice-related media coverage influences clinical practice	108 (84.4)
More detailed explanation of medical procedures	100 (78.1)
Requesting additional diagnostic tests considered clinically unnecessary	93 (72.7)
More frequent consultation requests	88 (68.8)
Greater emphasis on informed consent and patient information	80 (62.5)
Spending more time with patients	58 (45.3)
Preference for non-invasive approaches over invasive alternatives	57 (44.5)
Recommending hospital admission beyond strict clinical indications	45 (35.2)
Avoidance of patients perceived to have a high likelihood of litigation	43 (33.6)
Avoidance of treatments associated with a high risk of complications	31 (24.2)
Avoidance of patients with complex medical problems	27 (21.1)

Percentages represent the combined proportion of participants selecting “Agree” or “Strongly Agree” (*n* = 128).

**Table 5:** Factors associated with reconsidering medical decisions due to malpractice concerns.

Variable	Category	Reconsidered Decision, n/N (%)	*p*
Personal malpractice lawsuit experience	No	86/107 (80.4)	0.045 *
	Yes	12/21 (57.1)	
Colleague malpractice lawsuit experience	No	15/28 (53.6)	0.002 *
	Yes	83/100 (83.0)	
Awareness of defensive medicine	No	5/5 (100.0)	0.590 *
	Yes	93/123 (75.6)	
Adequate knowledge of defensive medicine	No	20/29 (69.0)	0.223
	Undecided	18/26 (69.2)	
	Yes	60/73 (82.2)	
Awareness of MMSS	No	70/90 (77.8)	0.651 *
	Yes	28/38 (73.7)	
Malpractice-related fear/anxiety	None	2/7 (28.6)	0.001
	Rarely	4/4 (100.0)	
	Sometimes	14/19 (73.7)	
	Frequently	33/50 (66.0)	
	Always	31/34 (91.2)	
Institution type	State hospital	15/17 (88.2)	0.347
	Training and research hospital	57/74 (77.0)	
	University hospital	26/37 (70.3)	
Annual patient volume	0–1000	14/17 (82.4)	<0.001
	1001–5000	37/37 (100.0)	
	5001–10,000	21/28 (75.0)	
	10,001–15,000	13/21 (61.9)	
	>15,000	13/25 (52.0)	
Academic title	Specialist physician	80/95 (84.2)	<0.001
	Assistant professor	5/14 (35.7)	
	Associate professor	13/19 (68.4)	
Age group	25–39 years	56/70 (80.0)	0.310
	40–49 years	28/41 (68.3)	
	50–59 years	14/17 (82.4)	
Professional experience	0–5 years	22/30 (73.3)	0.310
	6–10 years	15/15 (100.0)	
	11–15 years	36/44 (81.8)	
	16–20 years	20/34 (58.8)	
	>20 years	5/5 (100.0)	

* Fisher’s exact test was used where appropriate. For variables with more than two categories, Pearson chi-square test was used. MMSS: Medical Malpractice Stress Syndrome.

**Table 6:** Defensive medicine composite scores among emergency medicine physicians.

Score	Number of Items	Possible Range	Observed Range	Mean ± SD
Assurance Defensive Medicine Score	9	9–45	21–43	33.83 ± 5.21
Avoidance Defensive Medicine Score	4	4–20	4–19	11.00 ± 3.60

Higher scores indicate a greater tendency toward defensive medicine practices. Internal consistency was good for both the Assurance Defensive Medicine Score (Cronbach’s α = 0.776) and the Avoidance Defensive Medicine Score (Cronbach’s α = 0.775). SD; Standard deviation.

**Table 7:** Correlations between malpractice-related anxiety and defensive medicine composite scores.

Variable	Assurance Defensive Medicine Score	Avoidance Defensive Medicine Score
Malpractice-related fear/anxiety	r = 0.383	r = 0.139
*p* value	**<0.001**	0.118

Higher scores indicate a greater tendency toward defensive medicine practices. Internal consistency was good for both the Assurance Defensive Medicine Score (Cronbach’s α = 0.776) and the Avoidance Defensive Medicine Score (Cronbach’s α = 0.775).

## Data Availability

The datasets generated and/or analyzed during the current study are not publicly available due to privacy and ethical restrictions but are available from the corresponding author on reasonable request and with permission of the relevant ethics committee.

## Abbreviations

The following abbreviations are used in this manuscript:

MMSS	Medical Malpractice Stress Syndrome
DM	Defensive Medicine
ED	Emergency Department
SD	Standard Deviation
IRB	Institutional Review Board
CHERRIES	Checklist for Reporting Results of Internet E-Surveys
